# Attachment Style and Emotional Regulation as Protective and Risk Factors in Mutual Dating Violence among Youngsters: A Moderated Mediation Model

**DOI:** 10.3390/healthcare12060605

**Published:** 2024-03-07

**Authors:** Jessica Morales-Sanhueza, Guadalupe Martín-Mora-Parra, Isabel Cuadrado-Gordillo

**Affiliations:** Department of Psychology and Anthropology, Faculty of Education and Psychology, University of Extremadura, 06071 Badajoz, Spain; cuadrado@unex.es

**Keywords:** young adults, difficulties in emotional regulation, attachment styles, well-being, mutual violence

## Abstract

Violence in intimate partner relationships among young adults has become a global health problem given its prevalence and its negative effects on physical and psychological well-being. The severity of the problem has given rise to a large body of research that has attempted to find the variables associated with victimization in young couples (for example, attachment style, emotional regulation skills or empathy, among others). Moreover, traditionally, many of these investigations have only considered the point of view of female victims within a gender violence approach. However, in recent times, more and more evidence of the existence of mutual violence in young relationships has been found. These findings, combined with simplistic explanations of the phenomenon, have proven to be insufficient to prevent it. In this context, the main objective of this study was to investigate how some variables linked to dating violence interact with each other, modifying the mutual violence young people suffer and exercise. Considering this, different instruments were administered (the Experience in Close Relationships Scale (ECR-R); Difficulties in Emotional Regulation Scale (DERS-E); Basic Empathy Scale (BES); and Multidimensional Couple Violence Scale (EMVN)) to a sample of 557 young Chileans. The analysis of the results, based on the construction of a moderated mediation model, reveals that difficulties in emotional regulation are a predictor of violence in intimate partner relationships, whose direct and indirect effects on the violence exercised can be moderated by that partner’s attachment style. The findings also reveal that there is no association between empathy and violence, and they highlight that both men and women are victims and aggressors at the same time. This demonstrate the need to consider prevention and intervention strategies aimed at both sexes, since intimate partner violence is mutual and reciprocal.

## 1. Introduction

Intimate partner violence in young adults has become a highly relevant social health problem, with growing research interest due to its high prevalence in different countries around the world [1,2]. The interest in addressing this phenomenon in young people is crucial since, at this stage of life, the relationship patterns that determine the way young people will interrelate with others in the future are established [3]. In this context, it is important to highlight that intimate partner violence is any behavior causing physical, psychological, or sexual harm to one of the partners in an intimate relationship, regardless of sex, gender, or who plays the role of the victim or perpetrator [4,5].

The severity of violence in intimate partner relationships between young people is related to the continuity over time of these violent behaviors due to, generally, the fact that the first aggressions do not usually lead to a breakup and cause, in the long term, adverse effects on young peoples’ physical health as well as on their social and psychological well-being [6,7,8]. In fact, it is common for the partners to maintain the relationship, disregarding the abuse based on strongly held beliefs, ideas, or myths about romantic love. In this situation, many victims even justify violent behaviors by mistakenly confusing them with displays of an idealized romantic love [9,10]. These circumstances frequently lead to the normalization of aggressions, which are considered an inherent part of the relationship, or to strategies to cope with their couple conflicts, even minimizing or denying them, especially when these violent episodes are sporadic or conducted through social networks [11,12,13,14,15,16].

In relation to the prevalence of these violent behaviors in couples, women have traditionally been considered as the victims, a fact that may stem from sexist behaviors maintained by men, and by women themselves, who frequently demonstrate benevolent sexist attitudes [17]. Hence, many women involved in violent relationships consider that the role of men is to protect and care for them, placing themselves in a position of inferiority with respect to men, and behaving in accordance with sexist expectations [18,19,20]. However, currently, other research has pointed out that women also play the role of the aggressor, finding that the phenomenon of violence in young couple relationships affects both men and women in a reciprocal and bidirectional way [1,21,22,23,24,25].

Considering the above, several studies have analyzed the possible causes underlying the development of these abusive partner relationships and found that the causes are diverse. A theoretical review of 113 investigations identified 30 different variables related to violence in intimate partner relationships. This study allowed for the identification of relevant variables that seem to have an important effect on the establishment and maintenance of abusive relationships, such as the attachment style of the partners and their emotional regulation skills, as well as empathy [26].

Focusing attention on empathy, which is defined as the ability to understand and experience the emotions, thoughts and experiences of another person, which entails experiencing an emotional response and establishing an empathic connection to both emotional and cognitive experiences [27], it is cataloged as one of the most important precipitating factors in intimate partner violence, with the importance of its inhibitory role against violent impulses emphasized, considering that this variable is a protective factor that reduces the probability of aggression [28,29]. In contrast, a low level of empathy, or its absence, would increase the probability of a person assaulting their partner, becoming a facilitator of violence [28,29,30].

Another factor linked to dating violence among adolescents and young people is attachment style. Adult attachment styles are emotional and behavioral patterns that significantly influence how people engage in affective relationships and manage conflict within their intimate and romantic relationships. These styles are based on early attachment experiences in infancy and associated with abandonment anxiety and intimacy avoidance. Secure-attachment people trust their partner; they are comfortable with intimacy and have low levels of attachment anxiety and avoidance. On the other hand, people with a dismissing attachment style fear intimacy and dependence, are distant with their partners and present high levels of avoidance. Preoccupied-attachment people search for security and constant displays of affection from their partners, fear abandonment and present high levels of anxiety. Finally, fearful-attachment people present high levels of anxiety and avoidance, tending to engage in contradictory behaviors that alternate between anxious and avoidant attachment behaviors [31].

In this regard, several studies have highlighted a connection between secure attachments and the absence of violence in couple relationships. Likewise, secure attachment has been related to better mental and physical health, and, therefore, to well-being [32,33,34]. Instead, it has been documented that those with an insecure attachment style are more likely to suffer from physical diseases, as well as poor psychological and neurobiological functioning, which continues from childhood into adulthood [35]. Furthermore, many investigations have indicated that attachment styles characterized by high levels of anxiety and avoidance are significantly related to both aggression and victimization during physical violence in an intimate relationship. Thus, attachment style has a crucial role in the way emotions are managed in couples, while also being a moderating factor that could influence, in turn, their emotional regulation, as well as the processes of the functional and dysfunctional expression of anger [36,37,38,39,40,41].

Regarding the difficulty with emotional regulation, understood as the difficulty for a person to effectively manage their emotions or their inability to maintain emotional balance in times of stress, by not being able to control their impulsive reactions [42], this variable has also been associated with aggressive behaviors. In this way, several authors have indicated that aggressive attitudes are correlated with low levels of emotional regulation, while the absence of violent behavior is associated with greater emotional regulation. Likewise, they point out that difficulties in emotional regulation cause not only various health problems, but they also negatively affect cognitive processes and behavior, facts that can finally lead to the development of different psychopathologies [43,44,45,46].

Finally, sexual orientation refers to a person’s sexual attraction to men, women, or both. In general, people who are attracted to the opposite sex are considered heterosexual, while those who are attracted to the same sex, or both sexes, are considered non-heterosexual [47]. In this regard, although violence in intimate partner relationships can affect people of any sexual orientation, research focused on young people indicates that heterosexual couples have a higher prevalence of violence in both men and women [48,49,50,51], with this prevalence being similar in different countries. In this context, Chile is no exception, since there is evidence that indicates that violence among young Chileans is a problem equivalent (in magnitude) to what has been reported in Latin America and the rest of the world [2]. Specifically, the seriousness of this phenomenon in the country has been proven due to the high prevalence of violence in romantic relationships among young people (psychological violence 51% and physical violence 25%) [2], as well as the consequences derived from this phenomenon that threaten the well-being of young Chileans.

Despite the evidence found, few studies have focused on analyzing the variables that influence the initiation and maintenance of abusive dating relationships in Latin America, and the way those factors interact with each other, as they mainly focus on describing the prevalence and the factors associated with the phenomenon. Additionally, even fewer studies have focused on mutual violence in the dating relationships of young couples. Consequently, more specific studies are needed to analyze the influence and interactions of less-explored variables, such as difficulties in emotional regulation, attachment style, empathy, and sexual orientation, to determine their importance in the prediction of violence and in the promotion of well-being in young couples. In this context, the main objectives of this study are (i) to analyze the prevalence of mutual violence in young university students, and (ii) to study the influence that variables such as difficulties in emotional regulation, attachment style, or empathy have on the violence exercised or suffered in intimate partner relationships between young adult couples, analyzing, additionally, which variables are mediators and moderators. Considering this, the following hypothesis were posited:

**H1:**  *Men and women are both victims and perpetrators of violence in their intimate partner relationships.*

**H2:**  *Attachment styles are related to the violence exercised and suffered in intimate partner relationships.*

**H3:**  *Difficulty with emotional regulation is a predictor of violence in intimate partner relationships in young people, and its influence is also mediated by the attachment styles and empathy that the partners have.*

## 2. Materials and Methods

### 2.1. Sample

The sample size required was calculated using G*power 3.1 software’s [52,53] recommendations (statistical power = 80%; Effect size = 0.02; α = 0.05). The minimum sample size recommended by this software was 395. The final sample consisted of 557 participants, 171 of whom were men (30.7%) and 386 of whom were women (69.2%), aged between 18 and 29 years old (M = 21.62; SD = 2.44).

The sample was selected in three phases. In the first phase, the Universidad Católica de Temuco was chosen, considering the age of the students and the objectives of the study. The first phase ended after contacting the university to explain the characteristics of the research and to ask for permission to administer the questionnaires. The second phase consisted of selecting, randomly, some classes from different grades and majors. The researchers themselves went to every class to obtain informed consent and assure the anonymity and voluntary participation of the students. In each class, the questionnaires were administered collectively. Finally, the third phase consisted of determining the final sample based on the inclusion and exclusion criteria; the inclusion criteria were that participants were university students who had had a previous relationship and were no older than 29 years of age. Therefore, since all participants were university students, only students who had never been in a relationship and who were older than 29 years of age were excluded from the sample.

### 2.2. Instruments

The participants answered a brief survey with questions about their sociodemographic characteristics: age, biological sex, sexual orientation and gender identity, as well as the Spanish version of the Experiences in Close Relationships, the Spanish version of the Difficulties in Emotion Regulation Scale, the Spanish version of the Basic Empathy Scale and the Multidimensional Scale of Dating Violence.

#### 2.2.1. Experience of Close Relationships Scale (ECR-R)

This questionnaire is the Spanish version of the Experiences in Close Relationships (ECR). The Spanish version that was used in the research was a self-reporting instrument comprising a total of 36 items grouped in two Likert-type scales with 7 anchor points, where 1 means “totally disagree” and 7 means “totally agree”.

Thus, based on its avoidance and anxiety scales, the ECR-R makes it possible to establish 4 adult attachment styles depending on the level of avoidance and anxiety obtained: secure attachment; preoccupied; dismissing; and fearful [54].

The validation of the ECR-R questionnaire carried out by Alonso-Arbiol et al. in 2007; they performed an exploratory factor analysis with oblique rotation, obtaining two factors that explained 34.65% of the accumulated variance (avoidance 18.9% and anxiety 15.7%). Cronbach’s alpha was 0.87 for avoidance and 0.85 for anxiety [55].

#### 2.2.2. Difficulties in Emotion Regulation Scale (DERS-E)

This instrument corresponds to the adapted Spanish version of the Difficulties in Emotion Regulation Scale (DERS), developed by Gratz and Roemer in 2004. This instrument is a self-report questionnaire that allows for the evaluation of emotional regulation difficulties. The scale used is a Likert-type scale with five anchor points, in which 1 corresponds to “almost never” and 5 to “almost always”. Higher scores are indicative of a greater degree of difficulty with emotional regulation [56].

The review and adaptation of the DERS-E to the Chilean population, composed of 25 items, confirmed, in general terms, that it is an instrument with reliable and valid psychometric properties, presenting internal consistency indexes that fluctuated between 0.66 and 0.89 [57].

#### 2.2.3. Basic Empathy Scale (BES)

The original self-report scale (Basic Empathy Scale) consists of 20 items that allow for the individual or collective assessment of affective, cognitive and global empathy. This questionnaire includes a Likert-type scale with five anchor points (1 = totally disagree and 5 = totally agree) [58]. A review carried out in the Spanish adolescent population performed an exploratory factor analysis to validate this scale, checking the adequacy of the sample for performing an analysis by means of the Kaiser–Meyer–Olkin test (KMO = 0.83). Subsequently, items with communalities below 0.40 were followed, as well as those whose highest factor weight was below 0.32, those with weights above 0.32 in more than one factor, and those in which the difference between the highest factor weight and the next was below 0.15. In this way, only 9 of the original items were retained. The final factorial solution performed on the nine items revealed the existence of two factors that explain 34.7% of the variance.

#### 2.2.4. Multidimensional Scale of Dating Violence (EMVN)

The EMVN is a valid and reliable scale that measures violent behaviors in the dating relationships that are established among young people. It is a 32-item self-report scale made up of two subscales that measure the violence exerted and suffered by university student couples (with three dimensions: physical and sexual assault, behavioral control and psycho-emotional abuse, as a victim or as an aggressor). The questionnaire includes a Likert-type scale with five anchor points (1 = totally disagree and 5 = totally agree) and the higher the score, the more violence exerted or suffered. This is an instrument with reliable and valid psychometric properties; presenting internal consistency indices that fluctuate between 0.88 and 0.80, it stands out for its easy application, correction and interpretation [13].

### 2.3. Procedure

Prior to the distribution of the questionnaires, both the research objectives and the procedures, instruments and techniques used were checked and approved by the Bioethics and Biosafety Committee of the University of Extremadura (Spain) (Ref. 95/2023). Subsequently, authorization from the University was obtained and, after that, the potential participants were informed of the objectives, methods and mechanisms used to guarantee anonymity, as well as the confidentiality of their responses and the voluntary nature of the study.

Once informed consent was obtained from the university students, the researchers went themselves to every class at a fixed date and time to administer the questionnaires. A QR code and a link to access the questionnaires (that were administered through the Google Forms platform) were shared. This platform allowed the information to be collected and sorted quickly and easily, also allowing participants to complete the questionnaires using their mobile phones or laptops. The instructions given by the researchers were the same in every class. Additionally, we stayed in the class while the students filled out the different questionnaires to clarify any possible doubts they could have.

### 2.4. Analysis of Data

To obtain the final sample, a preliminary analysis was conducted to identify participants whose responses to the questionnaire met the inclusion criteria (having previously been in a relationship). In addition, the descriptive and correlational analyses of the different variables included in the sample (attachment, difficulties in emotional regulation, empathy and violence exercised and suffered) were carried out by means of calculating the mean and standard deviation, as well as Spearman’s correlation coefficient.

Thereafter, to verify the existence of an association between attachment styles, difficulties in emotional regulation and empathy, the chi-square test was used, as well as Cramer’s V statistic, to measure the strength of the association.

**Figure 1:**
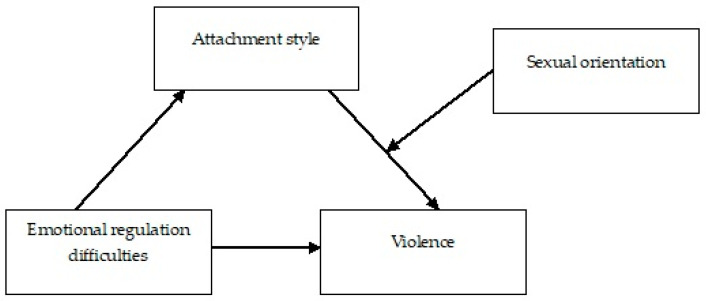
Serial mediation model 14.

Finally, a moderated mediation analysis was performed using “Process” V3.3 to determine the mediating effect of attachment style, as well as the moderating effect of sexual orientation, on the relationship between emotional regulation difficulties and violence in dating relationships [59] (Model 14) (Figure 1).

Process is an interface used in SPSS that employs least squares regression to estimate the significance and size of the direct and indirect effects in mediation models. With this tool, indirect effects are inferred using bootstrapping after generating an empirical representation of the sampling distribution of the indirect effects. Bootstrapping is suitable for linear hypotheses when the variables are not normally distributed [60]. All analyses were performed using the IBM SPSS v. 24 statistical package.

## 3. Results

### 3.1. Descriptive Statistics

The analysis of the results reveals that the sexual orientation of the participants was heterosexual (86%), homosexual (2.2%), bisexual (11.2%), pansexual (0.4%) and demi-sexual (0.2%). In relation to their gender orientation, 68.76% were female, 30.52% were male, 0.17% were fluid and 0.53% were non-binary.

The analysis also shows that all the men in the sample have exercised violence in their intimate partner relationships. On the other hand, the analysis reveals that 99.2% of the women have also committed violence towards their partners. Considering the frequency of violence, this analysis shows that 99.4% of men report being occasional aggressors, while only 6% acknowledge that they have committed violence on a frequent basis. On the other hand, 97.4% of women reported being occasional aggressors. Finally, 1.8% of women reported the frequent violence of their partners (Table 1).

In addition to the above, the data analysis revealed that 302 people in the sample (80 men and 222 women) said they were in a relationship at the time they participated in the study (54.3%).

In the case of the violence suffered, 100% of the men indicated that they had been victims of violence by their partners, 95.9% of them suffered occasional aggression and 4.1% frequent abuse. The responses of the women revealed that 99.2% of them had suffered violence in a dating relationship, with a frequency of occasional violence in 93.5% of the cases and frequent violence in 5.7% (Table 1). These results allow us to conclude that the subjects, regardless of sex, become aggressors and victims at the same time, thus revealing that dating relationships are characterized by mutual aggression and abuse.

Furthermore, considering the dimensions of violence, our analysis of the data reveals that, although violence is bidirectional, abusive behavior is different in men and women. Women experience a higher prevalence of psycho-emotional, physical and sexual violence. Men, on the other hand, exercise more physical and sexual violence, but experience more behavioral control than women (see Table 2).

Regarding their attachment style, the results revealed that 27.8% of the participants had a secure attachment, 21.5% had a dismissing attachment, 25% had a preoccupied attachment and, finally, 25.7% had a fearful attachment style. Focusing attention on sex, a higher prevalence of secure attachment was found in men (33.9%) compared to women (25.1%). In addition, the results show that women more often have a fearful attachment (27.2%) or preoccupied attachment style (25.6%) (Table 3).

Complementary to the above, our analysis of the results shows that 66.9% of those who state that they are not in a romantic relationship have a fearful attachment style. Likewise, 74% of those who say they currently have a partner have a secure attachment style (Table 4).

Furthermore, our analysis of the results reveals that 49.1% of the sample presented difficulties in emotional regulation. In relation to its prevalence by sex, 55% of women and 36% of men, respectively, present difficulties in emotional regulation. Likewise, 66.7% of those who have never used violence against their partners do not present difficulties in emotional regulation; on the other hand, 50.3% of those who have used violence in their intimate partner relationships have difficulty regulating their emotions. Added to the above, 87.2% of those who present insecure attachment styles present difficulties in emotional regulation (Table 5).

Finally, regarding empathy, the findings indicate that 46.65% of the sample have low levels of empathy and that women have higher levels compared to men (Table 6).

### 3.2. Association Analysis

As for the association analysis, a statistically significant association was found between the variable “difficulties in emotional regulation” (D.E.R.) and “attachment style” (χ^2^ = 345.378; *p* < 0.01), with the finding that the strength of the association, measured using Cramer’s V coefficient, is moderate (V = 0.455; *p* < 0.01). Likewise, a statistically significant relationship was found between “attachment style” and “exercised violence” (χ^2^ = 181.395; *p* < 0.01), with the strength of the association established as moderate (V = 0.329; *p* < 0.01).

On the other hand, a statistically significant relationship was found between “sexual orientation” and “exercised violence” (χ^2^ = 329.928; *p* < 0.01), with moderate associative strength (V = 0.386; *p* < 0.01), as well as between “attachment style” and “currently having a partner” (χ^2^ = 83.166; *p* < 0.01; V = 0.387; *p* < 0.01).

The analysis also reveals that there is no association between “sex” and “exercised violence or suffered” in intimate partner relationships (*p* > 0.05), between “sex” and “attachment style” (*p* > 0.05), between “empathy” and “exercised violence or suffered” (*p* > 0.05), or between “sex” and “emotional regulation difficulties” (*p* > 0.05).

Finally, our analysis of the correlation matrix (Table 7) indicated that “exercised violence” shows a positive correlation with “difficulties in emotional regulation” (D.E.R.).

Similarly, “exercised violence” correlates with “suffered violence”. Thus, young people who report having performed a high degree of violence against their partners show a high level of difficulty in regulating their emotions. These young adults also suffer a high degree of violence, being victimized themselves, both in the case of males and females. Additionally, a statistically significant and negative correlation was found between “attachment” and “D.E.R.”. This finding indicates that a secure attachment style is linked to fewer difficulties in emotional regulation. Finally, the variable “attachment” shows a negative correlation with “exercised violence”, revealing that secure attachment styles are related to less violence exercised in intimate partner relationships. In contrast, attachment styles characterized by high anxiety or avoidance are linked to the violence exercised and suffered in intimate partner relationships (Table 7).

### 3.3. Moderated Mediation Analysis

Two moderated mediation analyses were performed with 10,000 bootstraps, taking both exercised and suffered violence as the dependent variables. The coefficients of the moderated mediation model on exercised violence can be seen in Table 8.

The direct effect of “emotional regulation difficulties” on “attachment style” (Model 1) is statistically significant (β = −0.003; t = −12.29; *p* < 0.001).

Model 2 shows the effect of “emotional regulation difficulties”, “attachment style” and “sexual orientation” on “violence exercised “. In this regard, a direct effect of “emotional regulation difficulties” on “exercised violence” (β = 0.003; t = 2.69; *p* < 0.001) is found, as well as of “sexual orientation” on the degree of “exercised violence” in a dating relationship (β = 0.19; t = 3.74; *p* > 0.01). As for the interaction between “attachment” and “sexual orientation”, it is also statistically significant (β = −0.07; t = −3.28; *p* > 0.01).

Then, “sexual orientation” moderates the relationship between the style of “attachment” and the exercising of “violence” in dating relationships. The results have also shown that a difficulty with emotional regulation is a predictor of violence (R^2^ = 0.21) and mediated by the type of attachment.

A bootstrap procedure was used to evaluate the indirect effects and the confidence intervals (CIs). An indirect effect is significant if the CI does not include the value 0. For the pathway “difficulties in emotional regulation” → “attachment style” → “exercised violence,” a significant indirect effect, β = 0.0028; 95% CI [0.0012, 0.0045], was obtained.

Meanwhile, the coefficients of the moderated mediation model on violence suffered can be seen in Table 9.

The direct effect of “emotional regulation difficulties” on “attachment style” (Model 1) is statistically significant (β = −0.03; t = −12.29; *p* > 0.001). Therefore, “attachment style” has a partial mediating effect on “exercised violence”.

On the other hand, the effect of “emotional regulation difficulties”, “attachment style” and “sexual orientation” was analyzed in Model 2. The results reveal that these variables do not exert any statistically significant effect on “suffered violence”. Likewise, the interaction between “attachment styles” and “sexual orientation” is also not statistically significant (Table 9).

Finally, regarding indirect effects, for the pathway “emotional regulation difficulties” → “attachment style” → “suffered violence”, a significant indirect effect, β = 0.0028; 95% CI [0.0012, 0.0045], was obtained. Therefore, “attachment style” also exerts a partial mediating effect on “suffered violence”.

## 4. Discussion

The results found in this study indicate a high prevalence of violence exercised and suffered by young people in their intimate partner relationships, with a higher prevalence in the dimensions of behavioral control and psycho-emotional violence. Likewise, the findings reveal that both young men and women exercise and suffer violence, becoming victims and victimizers at the same time. Consequently, our first hypothesis is confirmed. In this way, the results are consistent with previous research, which has also highlighted that the aggressions that occur in relationships between young people can be mutual and reciprocal [2,21,22,23,24,25,48,49,50,51]. Moreover, it is important to highlight that, far from being a phenomenon confined to Latin America, more and more studies reveal similar results in different countries and places around the world [13,21,22,23,24,25].

However, these findings are not without controversy, due to the great tradition of research that has considered women as the only victims of violence in intimate partner relationships [50]. In this sense, the invisibility of the mistreatment that a man may receive from a woman is due to the smaller social repercussion that this entails. This fact can be seen, for example, in the social acceptance of certain physical aggressions committed by a woman against her male partner (for example, a slap in the face) when the latter has been offended. On the contrary, similar behaviors could hardly be considered non-harmful or unimportant if this type of aggression were committed the other way around. Thus, from this perspective, and in line with a patriarchal model, a woman could react violently towards a man without major social consequences [31,50].

On the other hand, in accordance with our second hypothesis, our results reveal interrelationships between violence and attachment styles. Thus, attachment styles characterized by high levels of anxiety and avoidance increase the risk of violence in intimate partner relationships. These results are consistent with those previously suggested by different researchers, indicating that people with insecure attachment styles may react aggressively towards their partners when they see their relationship threatened. Likewise, several studies have revealed that this reaction would occur mainly in those who present high anxiety about abandonment, since violent behavior would be used to demonstrate unsatisfied needs for closeness, while also becoming a maladaptive response to a possible abandonment by their partner [36,37,38,39,40,61,62,63,64,65].

The next relevant finding of this study confirms the interrelation between difficulties in emotional regulation and violence. In this sense, several authors have shown that difficulties in emotional regulation are a factor that hinders the prevention of violence [45,46,47,66,67]. In addition, it has been pointed out that people who present difficulties in emotional regulation do have greater difficulty in managing their negative emotions in stressful situations, especially among those with insecure attachment styles [46,61,62,63,64,65,66,67,68]. These studies are consistent with our findings, which show a high prevalence of insecure attachment styles in subjects with emotional regulation difficulties. These facts seem to explain the connection found between difficulties in emotional regulation, violence and the presence of insecure attachment styles [32,33,34,35,36,37,38,39,40,41,42,43,44,45,46,47]. Therefore, this combination of factors could translate into violent responses towards their partners when the aggressors feel that their attachment needs are not satisfied [68].

On the other hand, our analysis also reveals that there are no interrelationships between empathy and exercised or suffered dating violence. Consequently, our third hypothesis is partially confirmed, since the interaction between difficulties in emotional regulation, attachment style and empathy is not a key predictor of violence. These results are inconsistent with prior research that has highlighted the role of empathy, considering it a protective factor that reduces the probability of aggression [27,28,29,30]. A possible explanation of this fact could be related to the high prevalence of controlling behavior and psycho-emotional violence exercised and suffered by both young men and women. These kinds of conduct, which would mainly occur at present in a virtual context (for example, through social networks), where the levels of empathy generated are lower (especially considering that these interactions are mediated by a screen), may be the key to preventing empathy from exercising its protective role [16,27,28].

Finally, another relevant finding of our research is related to the construction of a moderated mediation model. Specifically, our analysis reveals that difficulties in emotional regulation, attachment styles and sexual orientation interact with each other, becoming moderating and modulating factors that modify the effect that these intersecting variables have on the precipitation of violent events. In this sense, the results show that difficulties in emotional regulation have a direct effect on the precipitation of violence. In addition, this effect seems to be mediated by a person’s style of attachment. Likewise, the results indicate that sexual orientation moderates the relationship between attachment styles and the perpetration of violence in dating relationships, with a partial mediating effect on the perpetration of violence. This analysis also determines that difficulties in emotional regulation are a predictor of violence in dating relationships.

Although the above results regarding the influence of difficulties in emotional regulation and attachment styles on violence were expected, given the evidence that had previously linked these variables to violence [41,42,43,44,45,46,47,61,62,63,64,65,66,67,68], the role of sexual orientation is noteworthy. In this regard, we can mention that, although violence in couple relationships can come from any person regardless their sexual orientation, recent studies focused on intimate partner relationships agree that heterosexual couples are characterized by a high prevalence of violence in both men and women [49,50,51]. This relational phenomenon could be linked to the normalization of the aggressive patterns of these partners due to an inadequate way of resolving conflicts, a situation that leads to the normalization of violence [48].

According to this, the influence of sexual orientation could be attributed to the fact that neither member of these couples identifies with gender-based violence. For example, many women justify their partners’ violent or abusive behaviors, misinterpreting them as signs of an idealized love influenced by benevolent sexism [17,69]. Likewise, some young women play the role of aggressors in their relationships by imitating the controlling and aggressive behaviors of young men. Furthermore, some studies have suggested that these girls may become victimizers by justifying their behavior based on female empowerment, which represents a rupture of the traditional gender paradigm in the context of more egalitarian relationships, such as the relationships between young university students [51].

Concurrently, many men may use violence to reaffirm their dominant position within their relationship, in accordance with the stereotypes of hostile sexism. At the same time, these young men may have a lower perception of victimization, not interpreting the violence they receive as severe due to a lower perception of the seriousness of these events or because the stereotypes of gender violence traditionally describe partner violence as exercised unilaterally by men [49,50,51,70,71,72,73,74]. Consequently, mutual violence in heterosexual young people could be traced back to a functionally violent and sexist family environment or to the context of increased social violence, such that their experiences during childhood and youth could have contributed to normalizing not only victimization, but also aggression, so that both men and women could consider abusive behavior as a normal way of interacting with their partner [75,76].

These findings have important practical applications, since the high prevalence of violence exercised and suffered by young people in their intimate relationships should be considered as a risk factor for their health, given its negative effects on physical and psychological well-being. Likewise, the existence of mutual violence in young people should lead to a new model of the interactions between aggression and victimization in violent couple relationships, introducing changes to the preventive measures adopted both at the political level and in health and educational contexts. Thus, the key to prevention would not lie in the design of programs aimed only at victimized women, but in the inclusion of men, understanding that both sexes can play the roles of aggressor and victim.

These prevention programs should be focused on the promotion of coeducation models from early childhood for both sexes, considering the variables traditionally linked to the phenomenon of dating violence (sexism, the normalization of violence, moral disengagement, etc.), but they should also incorporate work focused on fostering emotional regulation skills as an essential part of the development of coping strategies. Giving visibility to the importance of creating secure emotional bonds with parents and/or caregivers during early childhood will be a protective factor that will allow young men and women to maintain healthy interactions based on trust, security, and independence in their future relationships.

## 5. Conclusions

To date, the association between difficulties in emotional regulation and attachment styles in relation to the violence in young couple relationships has been poorly studied. However, it cannot be denied that these variables have a great relevance in the general well-being of young people. Furthermore, the dating violence phenomenon has become a source of mental and physical health problems for teenagers and young adults. In this context, one of the main contributions of this research is its integration of some of the less studied variables to understand the factors that contribute to violence in couple relationships. Likewise, this study succeeds in exploring the links between these variables by establishing how some of them may influence others, seeking complex explanations rather than focusing on simpler, unidirectional relationships.

Another major finding proves that difficulty with emotional regulation is a key factor in understanding the phenomenon of violence in dating relationships, since it was possible to determine both its association with and its direct and indirect effects on the violence exercised. Moreover, it was established that difficulty with emotional regulation is a predictor of violence, and that its role is mediated by the type of attachment style a person has. Likewise, sexual orientation moderates the relationship between attachment style and the perpetration of violence in dating relationships.

Our findings also allow us to conclude that young people, regardless of their sex, become aggressors and victims at the same time, thus revealing that dating relationships are characterized by a bidirectionality in partner violence, implying that both sexes mutually become the aggressors and victims of violence in intimate relationships, fostering and validating the normalization of aggressive behaviors, while being equally influenced by the network of relationships that we have established between the variables analyzed in this study. However, these findings must not be interpreted as an attempt to deny or minimize the existence of violence against women, but rather as a complementary finding which reveals the dynamics of the phenomenon of dating violence in a changing society.

Therefore, it is necessary to indicate that any attempt to prevent violence in intimate partner relationships, as well as to promote well-being and health in young people, should consider the incorporation of strategies that promote the development of emotional regulation from an early age and should be extended to both young men and women.

Finally, future lines of research should focus on LGTBI+ couples to explore in depth the phenomenon of violence, due to the high prevalence observed in these relationships [77,78,79,80], which highlights the need for us to better understand the factors that mediate and moderate the perpetration of intimate partner violence and understand the possible differences between heterosexual couples and LGTBI+ couples.

## 6. Limitations

The present study has some limitations that should be considered. First, it is a cross-sectional study, which makes it impossible to infer causality in this study. Longitudinal research could offer a complementary view of the findings provided by this study, and also allow us to observe the way in which emotional regulation difficulties and attachment styles evolve in terms of their influence on abuse in violent intimate partner relationships. Also, analyzing whether the bidirectionality of violence in couples is a phenomenon that is occurring across all types of abuse (physical, psychological and sexual) would help us to understand more deeply the mediating and modulating influence that attachment and emotional regulation exert on violence. In addition, when interpreting the results and implications of this study, it should be considered that the sample included only university students, so the results may not be representative of all young people. Therefore, studies that include samples of non-university students are recommended to extend the generalizability of these results.

## Figures and Tables

**Table 1:** Descriptive statistics: exercised and suffered violence.

	Exercised Violence		Suffered Violence	
Frequency	Men	Women	Men	Women
Never	0 (0%)	3 (0.8%)	0 (0%)	3 (0.8%)
Occasionally	170 (99.4%)	376 (97.4%)	164 (95.9%)	361 (93.5%)
Frequently	1 (0.6%)	7 (1.8%)	7 (4.1%)	22 (5.7%)

**Table 2:** Descriptive statistics: dimensions of violence exercised and suffered by men and woman.

Dimensions of Violence	Men	Woman
**Exercised Violence**		
Physical and sexual	14.6%	10.3%
Behavioral control	100%	99%
Psycho-emotional	73.6%	73.1%
**Suffered Violence**		
Physical and sexual	21.6%	32.1%
Behavioral control	100%	98.7%
Psycho-emotional	44.4%	46.8%

**Table 3:** Frequency of attachment styles in men and women.

Attachment Styles	Men	Women
Fearful	38 (22.2%)	105 (27.2%)
Preoccupied	40 (23.4%)	99 (25.6%)
Dismissing	35 (20.5%)	85 (22.0%)
Secure	58 (33.9%)	97 (25.1%)

**Table 4:** Frequency of attachment styles and a current partner.

Current Partner	Fearful	Preoccupied	Dismissing	Secure
No	95 (66.9%)	41 (29.5%)	78 (65.0%)	40 (25.8%)
Yes	47 (33.1%)	98 (70.5%)	42 (35.0%)	115 (4.2%)

**Table 5:** Descriptive statistics: attachment styles and difficulties in emotional regulation.

Attachment Style	Difficulties in Emotional Regulation
Fearful	38.3%
Preoccupied	31.8.%
Dismissing	17.1.%
Secure	12.8.%

**Table 6:** Descriptive statistics: empathy levels in men and woman.

Empathy Level	Men	Women
Low empathy	48.5%	44.8%
High empathy	51.5%	55.2%

**Table 7:** Spearman correlations.

	1	2	3	4	5
D.E.R.	-				
Attachment	−0.475 **	-			
Sexual Orientation	0.113 **	−0.058	-		
Exercised Violence	0.269 **	−0.343 **	0.056	-	
Suffered Violence	0.164 **	−0.190 **	−0.005	0.681 **	-

Note: 1 = difficulty with emotional regulation; 2 = attachment; 3 = sexual orientation; 4 = exercised violence; 5 = suffered violence; ** *p* < 0.01.

**Table 8:** Model of moderated mediation on violence exercised.

	Model 1. Attachment		Model 2. Exercised Violence	
	*Β*	T	*Β*	T
D.E.R.	−0.03 ***	−12.29	0.003 ***	2.69
Attachment			−0.012	−0.40
Sexual Orientation			0.19 **	3.74
Attachment × Sexual Orientation			−0.07 **	−3.28
R^2^	0.21		0.15	
F	151.16		24.43	

Note: ** *p* < 0.01; *** *p* < 0.001.

**Table 9:** Moderated mediation model on violence suffered.

	Model 1. Attachment		Model 2. Suffered Violence	
	*Β*	T	*Β*	T
D.E.R.	−0.03 ***	−12.29	0.002	1.26
Attachment			−0.05	−0.99
Sexual Orientation			0.09	1.13
Attachment × Sexual Orientation			−0.04	−1.23
R^2^	0.21		0.05	
F	151.16		7.57	

Note: *** *p* < 0.001.

## Data Availability

The data presented in this study are not available due to privacy reasons.
